# Depression and risk of incident heart diseases among older adults at CKM stages 0–3: evidence from the China Health and Retirement Longitudinal Study

**DOI:** 10.1080/07853890.2026.2690815

**Published:** 2026-06-26

**Authors:** Deling Zeng, Feifei Xu, Yanyuan Zhang, Lingxiao Zhou, Shurong Zhang, Yanqing Wang, Fengyi Duan

**Affiliations:** ^a^ Health Management Center, Sichuan Clinical Research Center for Cancer, Sichuan Cancer Hospital & Institute, Sichuan Cancer Center, University of Electronic Science and Technology of China, Chengdu, China; ^b^ Department of Critical Care Medicine, West China Hospital, Sichuan University, Chengdu, China; ^c^ Department of Anesthesiology, Sichuan Clinical Research Center for Cancer, Sichuan Cancer Hospital & Institute, Sichuan Cancer Center, University of Electronic Science and Technology of China, Chengdu, China; ^d^ Department of Endoscopy Center, Sichuan Clinical Research Center for Cancer, Sichuan Cancer Hospital & Institute, Sichuan Cancer Center, University of Electronic Science and Technology of China, Chengdu, China; ^e^ Department of Pediatric Oncology, Sichuan Clinical Research Center for Cancer, Sichuan Cancer Hospital & Institute, Sichuan Cancer Center, University of Electronic Science and Technology of China, Chengdu, China

**Keywords:** Depression, CESD, CKM, IHD, CHARLS

## Abstract

**Background:**

Evidence on the association between depression and incident ischemic heart disease (IHD) in individuals with cardiovascular kidney metabolic (CKM) syndrome is limited. This study investigated this association in older adults at CKM stages 0–3.

**Methods:**

Data was extracted from the China Health and Retirement Longitudinal Study (CHARLS). Participants meeting the criteria were included. Depression was defined as Center for Epidemiologic Studies Depression (CESD) scale ≥10. IHD mainly contained heart attack, coronary heart disease, angina, congestive heart failure. Association between depression and IHD was estimated in participants at CKM stage 0–3 using logistic regression models. The directed acyclic graph (DAG) was also used for variable selection. Association between depression and CKM stage 4 was also evaluated. Subgroup analyses (strata including sex, marital status, etc.), interaction p‑values and restricted cubic splines (RCS) were also conducted. Six sensitivity analyses were conducted, comprising using original data, inverse probability of treatment weighting (IPTW), treating the CESD score as both a categorical and continuous variable, etc. Simple mediation analyses were conducted to examine whether changes in BMI, CRP, and WBC mediated the depression‑IHD association.

**Results:**

4,738 participants were included. After multivariable adjustment, depression was significantly associated with an increased risk of IHD (OR 1.62, 95%CI:1.34–2.03) and CKM stage 4 (OR 2.43, 95%CI:1.62–3.66). Similarly, when variables were selected by DAG, the OR was 1.67 (1.34,2.07) for IHD and 2.45 (1.66,3.64) for progression to CKM stage 4. The trend persisted across the 6 sensitivity analyses. RCS revealed a linear relationship between CESD score and IHD risk, with consistent associations across subgroups and no significant mediation effects.

**Conclusion:**

Depression was prospectively associated with a higher risk of IHD among participants at CKM stages 0–3. This finding offers a new perspective for the management of IHD in the context of CKM syndrome.

## Introduction

Depression is a prevalent psychiatric illness whose core symptom is a prolonged and pervasive state of depressed mood [1,2]. The primary manifestations include diminished interest or pleasure in daily activities, coupled with notably compromised functional capacity [3]. The clinical symptoms can extend to include behaviors such as self-harm and suicide attempts, and in certain instances, may involve psychotic features like hallucinations or delusional thinking [4].

Data from the World Health Organization (WHO) estimates that around 5% of adults worldwide suffer from depression, indicating its extensive reach [1]. Furthermore, the number of individuals with depression in China experienced a sharp increase of about 25% between 1990 and 2017, reaching 56.36 million and representing over 20% of global cases [5]. Projections further suggest that by the year 2030, depression is expected to become the leading contributor to the global burden of disease, posing a significant challenge to public health and socioeconomic structures [6,7]. Emerging research continues to highlight the connections between depression and various physical illnesses [8–10]. Recently, the affection of depression to CKM has attracted increasing attention [11].

Cardiovascular-Kidney-Metabolic (CKM) syndrome was firstly proposed by Presidential Advisory from the American Heart Association (AHA) in October 2023. It was characterized by interconnected pathophysiological pathways containing metabolic dysregulation, chronic kidney disease (CKD), and impaired cardiovascular function. The resultant cross-organ system dysfunction significantly elevates the risk for major adverse cardiovascular events [12,13]. In recent years, CKM syndrome used to serve as a background state for studying how different biomarkers influence the risk of cerebrovascular diseases (CVD) and outcomes. For example, researchers proved that atherogenic index of plasma positively associated with the risk of stroke in individuals with CKM syndrome stages 0–3 [14,15]; another study also documented that an elevated cumulative triglyceride-glucose index was linked with an increased risk of stroke events in the population of early CKM stages [16]. Elevated metabolic score for insulin resistance was also demonstrated, increasing the stroke risk [17].

However, to date, research on the relationship between depression and CKM remains limited. The few existing studies primarily demonstrate an association between depression and CKM staging. A significant gap remains regarding the impact of depression on IHD risk among the population within the early CKM stages. In this study, we define IHD and investigate the longitudinal influence of depression on its incidence across these CKM stages (0–3), the relationship between depression and CKM progressing to stage 4 was also explored. This provides a novel perspective for analyzing and validating the relationships between depression, CKM, and IHD.

## Methods

### Study population

The China Health and Retirement Longitudinal Study (CHARLS) served as the data source for this analysis. Established in 2011, this nationally representative longitudinal survey collects information every two years from Chinese residents aged 45 years and older. Its content covers socio-demographics, health status, physical examinations, and laboratory biomarkers [18]. Participants were recruited using a multi-stage, stratified, probability-proportional-to-size sampling technique across numerous counties and provinces [18–20]. All data are publicly accessible online (http://charls.pku.edu.cn/en).

**Figure 1.**
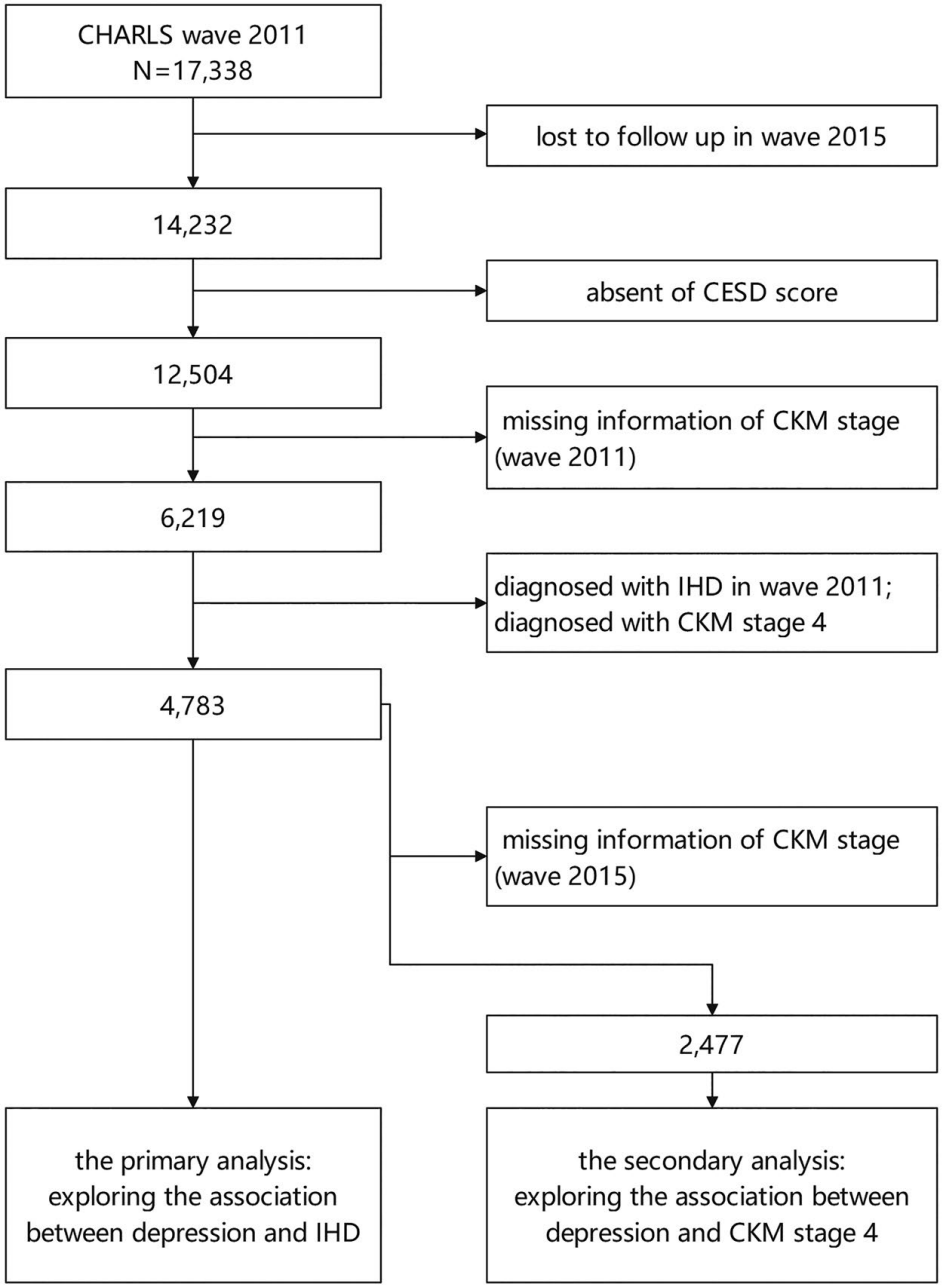
The flow chart of screening for enrolled individuals.

This study used data from the baseline (2011) and follow-up (2015) waves of the China Health and Retirement Longitudinal Study (CHARLS). Among the 17,338 initial participants, 4,738 met the inclusion criteria after excluding those who (1): were lost to follow‑up by 2015 (2); had missing CESD scale data (3); had incomplete information required for CKM staging (4); had a baseline diagnosis of IHD (5); had a baseline diagnosis of CKM stage 4. The detailed participant selection process is shown in Figure 1.

### Data collection and covariates

Variables in this study were derived from the following three components of the CHARLS survey: 1) interviewer-administered questionnaires, 2) physical measurements and performance tests, and 3) laboratory testing of blood samples.

Questionnaire-based variables included key sociodemographic factors: age, sex (male, female), education(primary school or below, middle school, college or above), marital status (with spouse, without spouse); health behaviors included: sleeping time, current cigarette use (no, yes), current alcohol use (> 1/month, ≤ 1/month, never), midday nap (no, yes); personal medical history containing hypertension (no, yes), diabetes (no, yes), arthritis (no, yes), falling down (no, yes); in this study, hypertension was defined as SBP >130 mmHg or DBP >80 mmHg or with self-reported diagnosis of hypertension or use of anti-hypertensive medications; diabetes was defined as fasting plasma glucose (FPG) ≥126 mg/dL or hemoglobin A1c (HbA1c) ≥6.5% or self‑reported physician‑diagnosed diabetes or use of glucose‑lowering medication; and relevant medications involving antihypertensive therapy, lipid-lowing therapy, glucose-lowering therapy, and antidepressants. Standardized physical examinations were conducted with trained staff. Physical measurement variables included blood pressure, height, weight (for calculating BMI), and waist circumference, etc. Fasting blood samples were collected for laboratory testing as previously described [21]. Relevant biomarkers for this analysis included levels of C-reactive protein (CRP), hemoglobin (Hb), white blood cell count (WBC), and platelet count, total cholesterol, high-density lipoprotein (HDL) cholesterol, low-density lipoprotein (LDL) cholesterol, triglycerides, fasting plasma glucose, glycated hemoglobin (HbA1c), and serum creatinine (for estimated glomerular filtration rate, eGFR).

### Definition of the key variables

The primary independent variable, depressive symptoms, were assessed using the 10-item Centre for Epidemiological Studies Depression Scale (CESD-10), a brief self-report measure designed to capture the frequency of such symptoms over the preceding week. This instrument has shown established reliability and sound psychometric properties within older adult populations in China. Respondents rate each of the 10 items on a four-point scale reflecting frequency, ranging from options such as “rarely or none of the time” to “most or all of the time”. Notably, items 5 and 8 are reverse-scored. The summed total score ranges from 0 to 30, with higher scores representing a greater severity of depressive symptomatology. A total score ≥10 was defined as indicating a depressive state, and a score < 10 was defined as normal [22]. This method has been widely used in CHARLS for exploring depression [23–25].

The outcome variable, IHD was defined by a self‑reported physician diagnosis in CHARLS (da007): “Has a doctor ever told you that you have heart disease (including heart attack, coronary heart disease, angina, congestive heart failure, or other heart problems)?” Participants who answered “yes” were considered as having the outcome, and were coded as 1, else were coded as 0.

Cardiovascular-kidney-metabolic (CKM) syndrome is categorized into five sequential stages, as outlined in a presidential advisory from the American Heart Association (AHA) [12]. Stage 0 encompasses individuals with no metabolic risk factors—such as overweight/obesity, abdominal obesity, hypertension, hypertriglyceridemia (triglycerides ≥ 135 mg/dL), prediabetes, diabetes, or metabolic syndrome—and without chronic kidney disease (CKD) or clinical/subclinical cardiovascular disease (CVD); Stage 1 includes individuals who have overweight/obesity, abdominal obesity, or prediabetes, but no other metabolic risk factors or CKD; Stage 2 comprises individuals with metabolic risk factors (e.g. hypertriglyceridemia, hypertension, diabetes, metabolic syndrome) and/or moderate- to high-risk CKD; Stage 3 is defined by the presence of subclinical CVD in addition to metabolic risk factors and/or CKD; Stage 4 includes individuals with self-reported clinical CVD alongside metabolic risk factors (e.g. hypertriglyceridemia, hypertension, diabetes, metabolic syndrome) and/or CKD [21]. In this paper, CKM progression was defined as reaching CKM stage 4. Detailed staging criteria are provided in Supplemental Table 1.

In the absence of direct clinical indicators for subclinical CVD, it was defined using risk equivalents. This included individuals with very high-risk CKD (Stages G4 or G5) as well as those whose 10-year CVD risk was 20% or higher. Predicted 10-year CVD risk was calculated using the American Heart Association’s PREVENT (Predicting Risk of CVD Events) Equations [26], which was conducted using the *preventr* package in R. CKM staging was determined based on the estimated glomerular filtration rate (eGFR), calculated *via* the Chronic Kidney Disease Epidemiology Collaboration (CKD-EPI) formula [27].

### Statistical analysis

Normality of continuous variables was assessed using the Shapiro–Wilk test. Continuous variables not normally distributed (*p* < 0.05) were presented as medians with interquartile ranges (IQR). Categorical variables were expressed in terms of frequencies and percentages. For comparisons between groups, the Mann–Whitney U test or Pearson’s chi-square test was employed as deemed appropriate. Missing data were handled using the *missForest* algorithm in R. Imputation was performed iteratively with maximum iterations = 15, number of trees per forest = 250.

The primary analysis was to assess the longitudinal relationship between depression (measured in 2011) and IHD (2015), two strategies were conducted. One was driven mainly by data and clinical knowledge, where clinically relevant variables and those with baseline differences between groups were entered stepwise into multivariable regression models. The analysis involved four sequentially adjusted models: Model 1 – unadjusted; Model 2 – adjusted for demographic variables (age, sex, marital status, education, sleep duration, current cigarette use, and alcohol use); Model 3 – further adjusted for laboratory measures (hemoglobin, white blood cell count, platelet count, and C-reactive protein); Model 4 – fully adjusted for demographic factors, laboratory parameters, clinical comorbidities (including hyperuricemia, arthritis, pulmonary diseases, sarcopenia, gastrointestinal diseases, hepatic disorders, and history of falls) and relevant medications (antihypertensive therapy, lipid-lowing therapy, glucose-lowering therapy, and antidepressants). To further examine this association, subgroup analyses were performed based on the fully adjusted model across variables including age, sex, marital status, and lifestyle factors such as current cigarette use, alcohol use, and midday nap. The other strategy was based on a directed acyclic graph (DAG). The minimal sufficient adjustment sets were selected for the regression analysis (Model 5). Meanwhile, the association between depression and the CKM stage 4 in wave 2015 was also analyzed as the secondary analysis.

The stability of our findings was evaluated through a series of five sensitivity analyses (SA). The specific approaches were as follows: In SA1, analysis was performed using the original dataset before data imputation to fitting the fully adjusted logistic regression model. SA2 employed inverse probability of treatment weighting (IPTW), with propensity scores estimated from all covariates in Model 4; To reduce the influence of extreme weights, the IPTW weights were trimmed at the 1st and 99th percentiles: values below the 1st percentile were set to the 1st percentile value, and values above the 99th percentile were set to the 99th percentile value, with no observations excluded. Balance was assessed by standardized mean differences (SMD); all covariates had post‑weighting SMD < 0.10, indicating adequate balance (see supplemental Table 2). For SA3, the CESD score was categorized into tertiles (three-level categorical variable), while for SA4 it was modeled as a continuous variable. In SA5, the cardiovascular risk assessment component (the PREVENT equations) was removed, and CKM staging based only on measured metabolic/renal criteria) to test robustness. Because the proportion of missing values exceeded 25%, two key possible confounders – physical activity and living area (urban/rural) – were included in the SA6 based on the fully adjusted model (model 4).

The potential for a nonlinear relationship of the CESD score with IHD was evaluated using restricted cubic spline (RCS) analysis with three knots (at the 10th, 50th, and 90th percentiles of CESD score). The median CESD score served as the reference. To enhance the robustness of our findings, RCS was conducted based on data‑driven approach (Model 4) and a DAG‑driven approach (Model 5). Analysis was conducted using the *rms* package in R.

Mediation analyses were performed using the mediation package in R to assess whether changes in BMI, WBC, and CRP (from baseline to follow-up) mediated the depression-IHD association. Because the outcome (IHD) was binary, a logistic regression model was fitted for the outcome equation in the mediation analysis, and mediator models were linear regression (change variables). The mediation effects (ACME, ADE, total effect) were estimated using the mediation package in R and are reported on the log‑odds scale.

Stata 16.1 (Stata Corp, College Station, TX, USA) and R 4.3.1 (R Foundation for Statistical Computing, Vienna, Austria) were employed for data analysis. Results were considered statistically significant if the two-tailed *p*-value was below 0.05.

## Results

### Baseline characteristics of participants

This study ultimately enrolled 4,738 eligible participants, who were then stratified into depression (*n* = 3,099) and non-depression groups (*n* = 1,684) based on diagnostic criteria. Table 1 summarizes the baseline demographics and clinical characteristics. The cohort comprised 2,552 females and 2,231 males. Compared with the non-depression group (median age: 57.00 years), individuals with depression were slightly older (median age: 58.00 years) and reported shorter sleep duration. Additionally, they exhibited a higher burden of comorbidities such as arthritis, pulmonary diseases, sarcopenia, and digestive disorders (Table 1). During follow‑up, participants in the depression group had a significantly higher proportion of progression to CKM stage 4 compared with the non‑depression group (7.9% vs. 3.4%, *p* < 0.001). Similarly, the incidence of IHD was also significantly higher in the depression group (11.1% vs. 7.0%, *p* < 0.001). Missing value summary for study variables was displayed in supplemental Table 3.

**Table 1.** The baseline characteristics of the participants in CHARLS wave 2011.

Variables	Depression		*p* value
	no	yes	
**n**	3099	1684	
**Age**	57.00 [50.00, 63.00]	58.00 [52.00, 64.00]	<0.001
**Sex (%)**			<0.001
female	1484 (47.9)	1068 (63.4)	
male	1615 (52.1)	616 (36.6)	
**Marital status (%)**			<0.001
with spouse	2764 (89.2)	1384 (82.2)	
without spouse	335 (10.8)	300 (17.8)	
**Sleeping time, hours**	7.00 [6.00, 8.00]	6.00 [4.00, 7.00]	<0.001
**Nap (%)**			<0.001
no	1363 (44.0)	862 (51.2)	
yes	1736 (56.0)	822 (48.8)	
**Current Cigarette use (%)**	2081 (67.2)	1257 (74.6)	<0.001
no	1018 (32.8)	427 (25.4)	
yes			
**Current Alcohol use (%)**			<0.001
>1/month	899 (29.0)	335 (19.9)	
**≤**1/month	261 (8.4)	134 (8.0)	
never	1939 (62.6)	1215 (72.1)	
**Hb, g/L**	144.00 [131.00, 157.00]	141.00 [129.00, 153.00]	<0.001
**Platelet, ×10^−9^**	205.00 [162.00, 252.00]	209.00 [163.00, 257.00]	0.076
**WBC, ×10^−9^**	6.00 [5.00, 7.20]	5.90 [5.00, 7.20]	0.398
**CRP, mg/dl**	1.04 [0.57, 2.02]	0.99 [0.53, 2.04]	0.272
**Falling down (%)**			<0.001
no	2739 (88.4)	1290 (76.7)	
yes	359 (11.6)	392 (23.3)	
**Hyperuricemia (%)**			0.038
no	2924 (94.4)	1613 (95.8)	
yes	175 (5.6)	71 (4.2)	
** Arthritis (%)**			<0.001
no	2229 (71.9)	886 (52.6)	
yes	870 (28.1)	798 (47.4)	
**Pulmonary disease (%)**			<0.001
no	2895 (93.4)	1490 (88.6)	
yes	203 (6.6)	191 (11.4)	
**Sarcopenia (%)**			<0.001
no	2828 (91.3)	1429 (84.9)	
yes	271 (8.7)	255 (15.1)	
**Digestive disease (%)**			<0.001
no	2526 (81.5)	1156 (68.6)	
yes	573 (18.5)	528 (31.4)	
**Hepatic diseases (%)**			0.001
no	3001 (96.8)	1598 (94.9)	
yes	98 (3.2)	86 (5.1)	
**Education**			<0.001
primary school or below	1940 (62.6)	1309 (77.7)	
middle school	1116 (36.0)	363 (21.6)	
college or above	43 (1.4)	12 (0.7)	
**Antihypertensive therapy**			0.089
no	2553 (82.4)	1353 (80.3)	
yes	546 (17.6)	331 (19.7)	
**Lipid-lowering therapy**			0.142
no	2964 (95.6)	1594 (94.7)	
yes	135 (4.4)	90 (5.3)	
**Glucose-lowing therapy**			0.048
no	2999 (96.8)	1610 (95.6)	
yes	100 (3.2)	74 (4.4)	
**Antidepressants**			<0.001
no	3089 (99.7)	1663 (98.8)	
yes	10 (0.3)	21 (1.2)	

Hb: hemoglobin; WBC: white blood cells; CRP: C-reactive protein; IHD: incident heart diseases; CKM: cardiovascular kidney metabolic syndrome; IHD: incident heart diseases.

### Prospective association of depression with CKM stage 4 or IHD among participants at CKM stages 0–3

**Figure 2.**
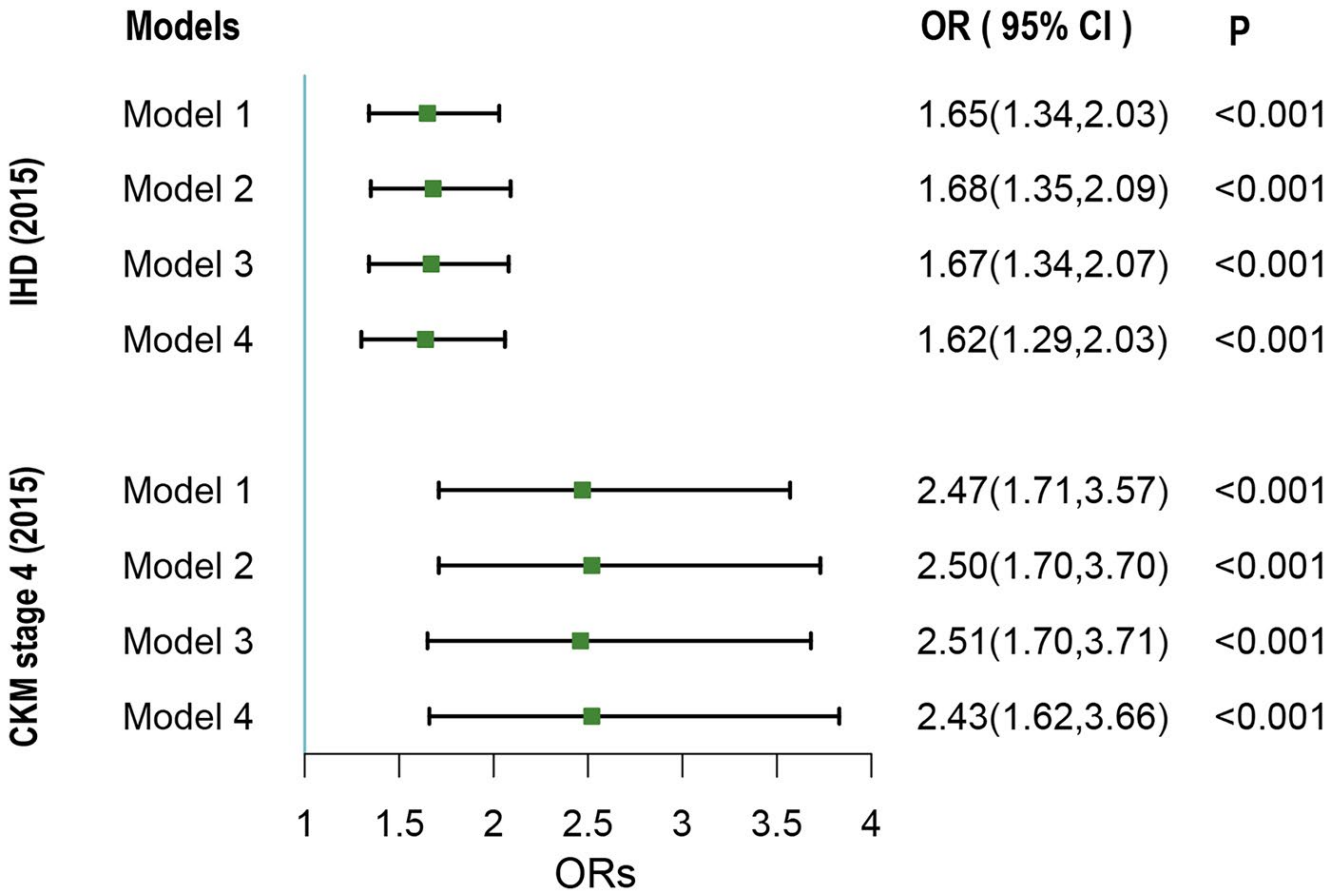
The forest plot for illustrating the association between the depression and CKM stage 4/IHD Model 1: crude model; Model 2: adjusted for age, sex, marital status, education, sleep duration, smoking, and alcohol use; Model 3: further adjusted for hemoglobin, white blood cell count, platelet count, and C-reactive protein; Model 4: further adjusted for demographic factors, laboratory parameters, clinical comorbidities and relevant medications; For models 1–4 using IHD as the outcome, the sample size was 4,783; for models using CKM stage 4 as the outcome, the sample size was 2,477.

**Figure 3.**
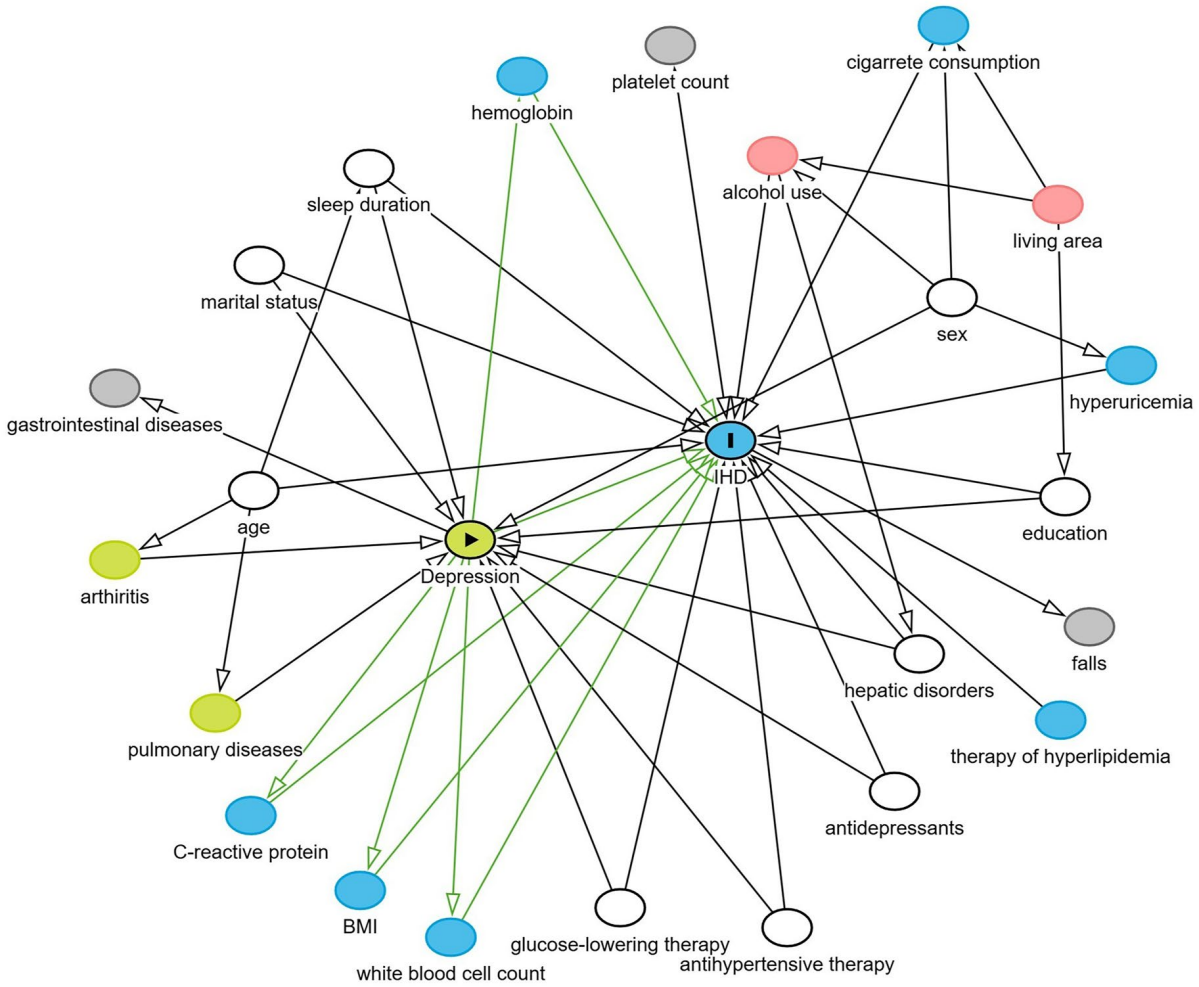
Directed Acyclic Graph (DAG) with depression as the exposure and incident heart diseases as the outcome.

Logistic regression analyses examined the association between depression (assessed in 2011) and subsequent CKM stage 4 or IHD (wave 2015) among participants initially at CKM stages 0–3. In the results of data-driven strategy, a significant positive association was found between depression and IHD across four adjusted models. The odds ratios (ORs) of model 1 (crude model) was 1.65 (1.34, 2.03), model 2 (adjusted for age, sex, marital status, education, sleep duration, current cigarette use, and alcohol use) was 1.68 (1.35, 2.09), mode 3 (further adjusted hemoglobin, white blood cell count, platelet count, and C-reactive protein) was 1.67 (1.34, 2.07), and mode 4 (further adjusted hyperuricemia, arthritis, pulmonary diseases, sarcopenia, gastrointestinal diseases, hepatic disorders, and history of falls) was 1.62 (1.29, 2.03); all *p*-values were < 0.001. Similarly, depression was significantly associated with a higher risk of progression to CKM stage 4 in this population, with the ORs 2.47 (1.71, 3.57), 2.50 (1.70, 3.70), 2.51 (1.70, 3.71), 2.43(1.62, 3.66) in the four models, respectively. All *p* values were less than 0.001. These results suggest that individuals with depression are at an increased risk of progressing to CKM stage 4 or developing IHD (Figure 2). In the results of DAG-drive strategy, a DAG was plotted, and the minimal sufficient adjustment sets including age, sex, marital status, education, sleep duration, hepatic disorders, antihypertensive therapy, antidepressants, and glucose-lowering therapy (Figure 3). After these covariates adjusted, the positive association still hold between depression and IHD (OR:1.67, 95%CI:1.34–2.07, *p* < 0.001) or CKM stage 4 (OR:2.45, 95%CI:1.66–3.64, *p* < 0.001), which strengthened the robustness of the analysis.

### Results of the sensitivity and subgroup analyses

Sensitivity analysis was performed to evaluate the robustness of the positive association between depression and IHD (Table 2). In SA1 (using the original data before random forest imputation), the risk of IHD remained significantly elevated, with an OR of 1.62 (95% CI: 1.29–2.04). After IPTW, all SMD were less than 0.1, which indicates an acceptable balance between the two groups (supplementary tale 2). A similar trend was observed in the IPTW-adjusted logistic regression (SA2), with an OR of 1.59 (1.38, 1.84). In SA3, when CESD scores were categorized into tertiles (cut-offs: 4 and 10), the highest tertile (Q3, CESD > 10) showed a significantly increased risk of IHD. Even when CESD was analyzed as a continuous variable (SA4), the positive association persisted (OR: 1.04, 95% CI: 1.02–1.05). In SA5, CKM was staged based only on measured metabolic/renal criteria, and the results showed the same trend with significance (OR: 1.76, 95% CI: 1.38–2.24, *p* < 0.001). After further adjusted living area and physical activity, the positive association still existed in SA6 (OR: 1.62, 95% CI:1.05–2.51, *p* = 0.03).

**Table 2.** The results of sensitive analysis.

Analysis	OR (95% CI)	*p* value
**SA1**	1.62 (1.29,2.04)	<0.001
**SA2**	1.59 (1.38,1.84)	<0.001
**SA3**		
Q2	1.03 (0.77,1.38)	0.854
Q3	1.65 (1.23,2.22)	0.001
**SA4**	1.04 (1.02,1.05)	<0.001
**SA5**	1.76 (1.38,2.24)	<0.001
**SA6**	1.62 (1.05,2.51)	0.030

SA1-SA5 were based on the full-adjusted model (model 4). SA1: using data before imputation; SA2: regression after IPTW; SA3: CESD as tertiles; SA4: CESD as continuous; SA5: CKM staged without the risk component; SA6: further adjusted with more variables including region and living area, based on model 4. SA: sensitivity analyses.

**Figure 4.**
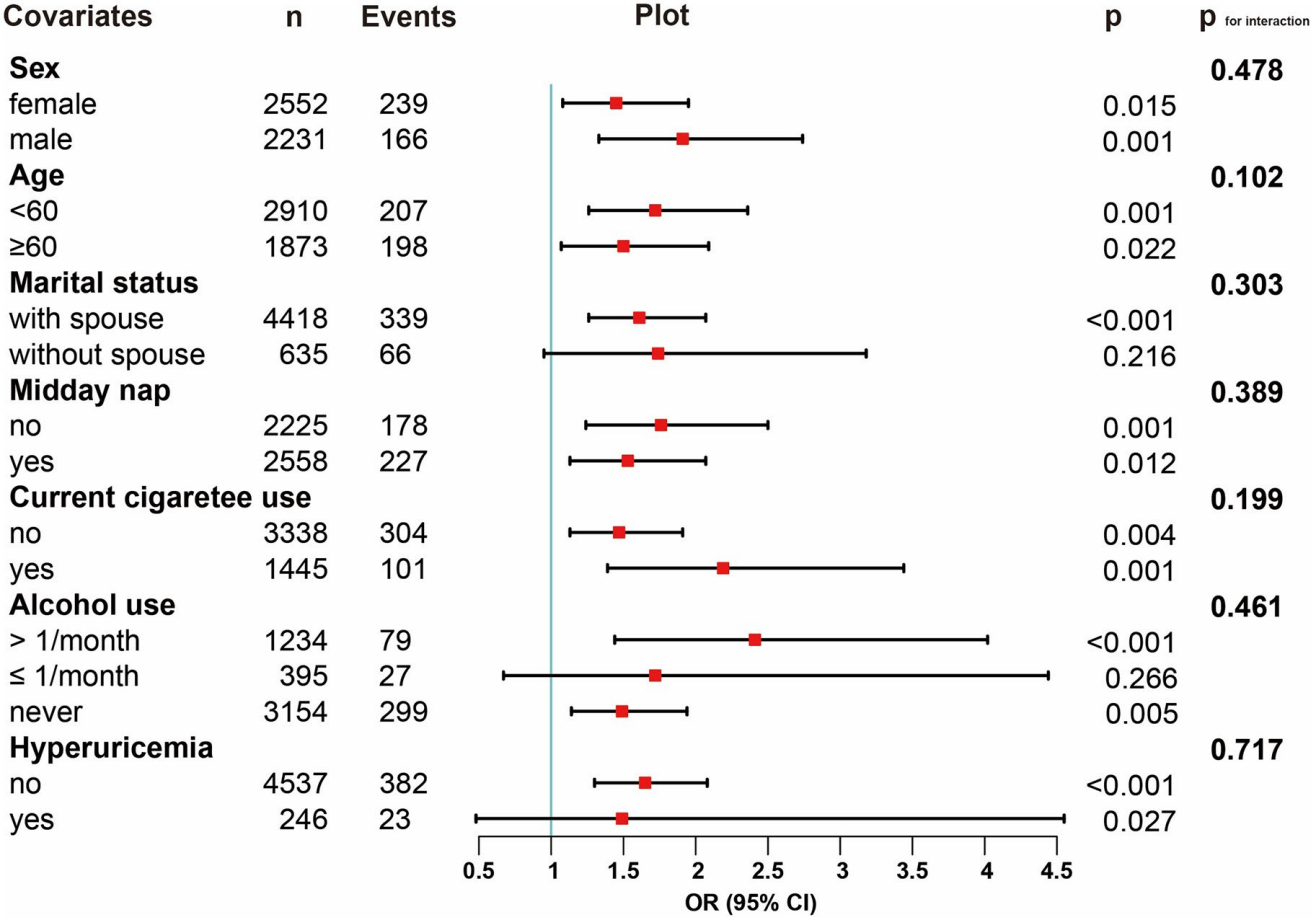
The forest plot of the subgroup analysis for estimating the association between depression and IHD.

Subgroup analyses across strata such as age, sex, alcohol use, and current cigarette use consistently showed elevated ORs in most subgroups. However, all interaction p-values exceeded 0.05, indicating no significant effect modification by these covariates (Figure 4). The findings further support the stability of the observed association.

Overall, the sensitivity and subgroup analyses confirmed the robustness of the relationship between depression and IHD.

### The nonlinear relationship between CMI and hyperuricemia reflected by RCS

**Figure 5.**
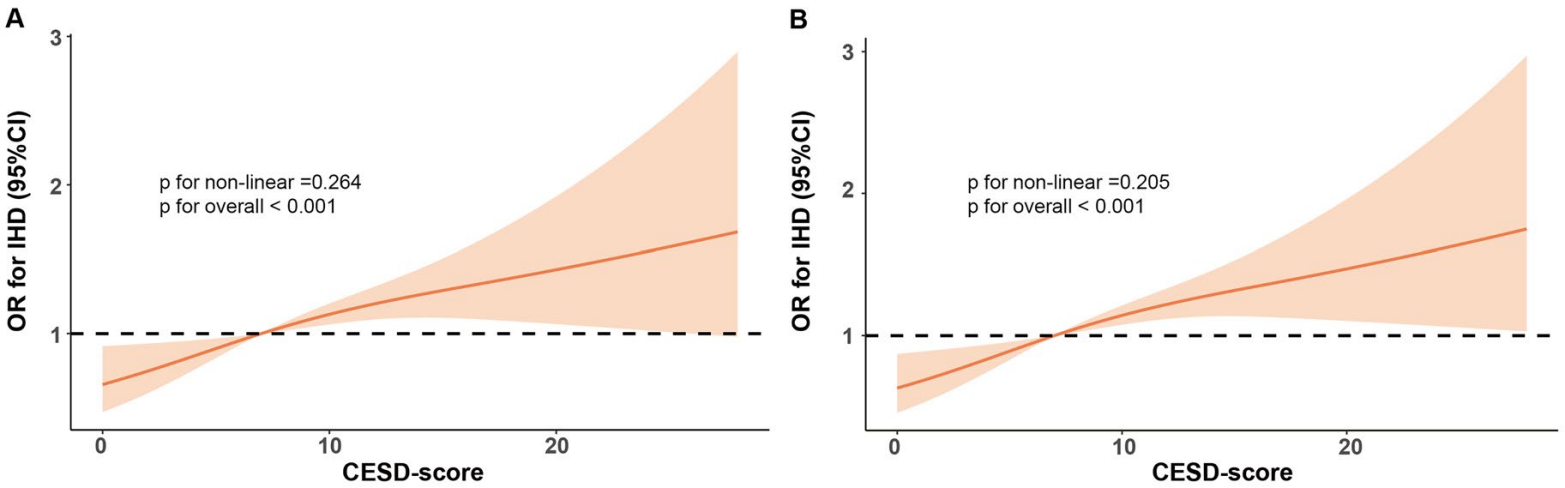
RCS displaying the nonlinear relationship between the CESD score and IHD A: based on data-driven strategy (model 4); B: based on the DAG-driven strategy (model 5. Three knots were placed at the 10th, 50th, and 90th percentiles of CESD score (values: 1, 7, 17). The reference value (OR = 1) was set at the median CESD score. Shaded area represents 95% confidence interval. Most participants (90%) had CESD scores between 0 and 21.

Restricted cubic splines were used to examine the potential nonlinear association between the CESD score and IHD. As shown in Figure 5, the relationship did not demonstrate significant nonlinearity in either the data-driven and DAG-driven strategies. However, the overall association was statistically significant (both *p* for overall < 0.001), suggesting that the degree of depression may increase linearly with higher IHD risk.

### Mediation analysis of the association between depression and IHD

**Figure 6.**
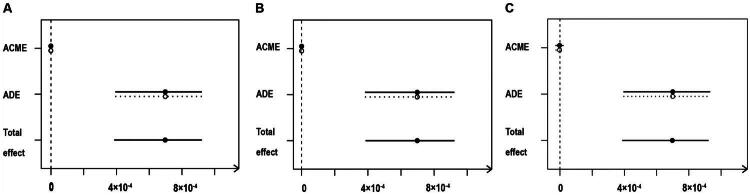
The results of the mediation effect analysis A, B, C displayed the mediative effect of the changes in BMI, WBC, and CRP, respectively.

The average causal mediation effects through changes in BMI, white blood cell count (WBC), and CRP were not statistically significant (Figure 6). These findings indicate that none of these potential mediators significantly mediated the association between depression and IHD.

## Discussion

The findings of this study revealed that depression is prospectively associated with increased risk of IHD as well as progression to CKM stage 4 in Chinese individuals aged over 45. RCS analysis indicated a linear relationship between the CESD score and IHD. Both sensitive analysis and subgroup analysis reaffirmed the robustness of the findings. These results substantiate the important role of depression in the development of IHD and suggest its potential as a target for IHD prevention or treatment. This research also identified waist and BMI as mediators in the effect of depression on IHD. To our knowledge, this is the first study to report a positive association between depression and IHD in participants at CKM stage 0–3.

Depression has been established as an independent risk factor for the development and poor prognosis of cardiovascular diseases (CVD) [28]. The association involves complex bidirectional pathophysiological mechanisms, including dysregulation of the autonomic nervous system, hypothalamic-pituitary-adrenal (HPA) axis hyperactivity, increased inflammation, and platelet aggregation [29,30]. This comorbid relationship significantly elevates morbidity and mortality, highlighting the necessity of integrated clinical management that addresses both mental and cardiovascular health in affected patients. On the other hand, since the CKM syndrome was defined by AHA in the October 2023, a large number of studies investigating predictors (or biomarkers) of cardiovascular and cerebrovascular diseases within the CKM framework have emerged [31–33]. Except that, depression has been linked to increased mortality in CKM syndrome, and managing depressive symptoms may reduce mortality risk for individuals at all stages of CKM [34]. To date, however, longitudinal evidence linking depression to the risk of IHD as well as CKM progression remains absent. Our analyses demonstrated that individuals with depression exhibited a 1.62-fold increased risk of IHD in the fully adjusted assessment. This result was strengthened by the DAG-directed minimal sufficient adjustment with an OR of 1.67 (1.34,2.07). Besides, the risk of CKM stage 4 was elevated to 2.43 times compared to participants without depression. This elevated risk persisted even after rigorous adjustment for potential confounders using IPTW methods, with the adjusted OR remaining stable at approximately 1.59(1.38,1.84). The consistency of these estimates across different analytical approaches reinforces the reliability of our findings. These results align partially with prior research conducted in middle-aged and older Chinese population, which identified that screening and management of depression among individuals with hypertension are essential for the primary prevention of CVD and premature death [35].

In this study, the robustness of the models was further tested by treating CESD score both continuously and as a three-category variable, with consistent results obtained across these specifications. Collectively, our results substantiate a positive prospective relationship between depression and IHD in the participants with CKM stages 0–3, attesting to the reliability and stability of this conclusion. Notably, a recent study partially aligns with our results, identifying higher depression severity as an independent predictor of CKM progression and mortality, which underscores its clinical prognostic value [23]; however, the specific relationship with IHD remained unexplored. Our study thus strengthens the evidence linking depression to CKM and further uncovered a positive association between depression and IHD in populations with early CKM. Further supporting our observations, depression exhibits a linear relationship with increased CVD risk among arthritis patients, and systemic inflammation appears to selectively potentiate this risk association in individuals with depression [24].

According to the current research, depression and heart disease engage in a mutually reinforcing cycle, with depression significantly hastening disease onset and progression [36]. Our study also revealed a linear relationship between depression and IHD by RCS with four knots. The underlying mechanism may lie as follows: Clinical evidence indicates that depression exhibits a linear association with an elevated atherogenic index of plasma (AIP), which contributes to a heightened risk of atherosclerosis (AS)—a key determinant of subsequent cardiovascular morbidity [37,38]. Besides, depression is often accompanied by the activation of central inflammatory responses, which have shown that inflammation plays a significant role in CVDs—including IHD, AS, MI, and HF [39,40]. Mechanism such endothelial dysfunction and platelet activation, lipid metabolism disorder, etc. are suggested as possible mechanisms that explaining the effect of depression to IHD [36]. This complex interplay of mechanisms may synergistically contribute to the elevated susceptibility to IHD observed in patients with depression.

Given the well‑established associations of depression and CVD with body weight and inflammation, our directed acyclic graph (DAG) suggested that changes in BMI, CRP, and WBC might serve as potential mediators of the depression‑IHD relationship. We therefore conducted mediation analyses using change‑based mediators. However, none of the indirect effects reached statistical significance. This finding suggests that, in our study population, the effect of depression on incident CVD is not substantially mediated through changes in these anthropometric or inflammatory markers.

In general, we revealed depression is positively associated with the prevalence of IHD in the middle aged and elderly Chinese with CKM stages 0–3. Community-based depression screening could potentially contribute to a reduction in IHD incidence. Moreover, such an approach could provide valuable early warning of cardiovascular risk for individuals with undiagnosed depression, prompting greater attention to both psychological and somatic health and encouraging timely intervention.

However, our study is still limited by several factors. First, depression was assessed using brief self-reported questionnaires, which may introduce some measurement bias compared with more comprehensive, clinician-administered diagnostic evaluations. However, the CESD scale employed in this study has been widely validated and is commonly used in epidemiological research. Second, the construction of the CKM was adapted from existing literature with certain modifications, which may affect the generalizability of the model to other populations or contexts. Third, the study focused on Chinese adults aged 45 years and above; therefore, the findings may not be fully generalizable to other ethnic groups or younger populations, and further validation in diverse global populations is warranted. Finally, the analysis was based on a retrospective observational design; logistic regression can not account for the timing of events or varying follow‑up durations, future prospective clinical studies are needed to address the time-to-event analyses and to strengthen the robustness of the conclusions.

## Conclusion

In this nationally representative cohort of older Chinese adults, depression was prospectively associated with a higher risk of IHD among participants at CKM stages 0–3. This finding provides a new perspective for the prevention and management of IHD within the CKM syndrome framework.

## Supplementary Material

supplementary files.docx

figure legend.docx

## Data Availability

The data will be available on reasonable request, and the datasets analyzed in this study are publicly available from the official CHARLS repository at: http://charls.ccer.edu.cn/charls/ [18].
