# Supplementary material for: Depression and risk of incident heart diseases among older adults at CKM stages 0–3: evidence from the China Health and Retirement Longitudinal Study
Source: Ann Med. 2026 Jun 26;58(1):2690815. doi: 10.1080/07853890.2026.2690815 (PMC13312823; doi:10.1080/07853890.2026.2690815)
Supplement: supplementary files.docx [file IANN_A_2690815_SM2144.docx]

**Supplemental Table 1 Definitions of CKM syndrome stages adapted for CHARLS data**

| **CKM stages** | **Definition** |
| --- | --- |
| **Stage 0: No CKM risk factors** | "Individuals without metabolic risk factors (overweight/obesity, abdominal obesity, hypertension, hypertriglyceridemia, prediabetes, diabetes, MetS), CKD or subclinical/clinical CVD.  All criteria are met：  ● BMI< 23 kg/m^2^  ● Waist circumference <80/90 cm in female/male  ● SBP <130 mmHg and DBP <80 mmHg without self-reported diagnosis of hypertension or use of anti-hypertensive medications  ● TG < 135 mg/dL  ● Fasting blood glucose < 100 mg/dL and HbA1c < 5.7% and without self-reported diagnosis of diabetes, use of insulin, or oral hypoglycemic agents  ● No MetS  ● eGFR ≥ 60 ml/min/1.73m^2^  ● Predicted 10-year CVD risk < 20%  ● No clinical CVD |
| **Stage 1: Excess or dysfunctional adiposity** | Individuals with overweight/obesity, abdominal obesity, or dysfunctional adipose tissue, without the presence of other metabolic risk factors or CKD  Any of the three criteria is met:  ● Overweight/obesity  ● Abdominal obesity  ● Prediabetes  All criteria are met:  ● SBP <130 mmHg and DBP <80 mmHg without self-reported diagnosis of hypertension or use of anti-hypertensive medications  ● TG < 135 mg/dL  ● Fasting blood glucose < 126 mg/dL and HbA1c < 6.5% and without self-reported diagnosis of diabetes, use of insulin, or oral hypoglycemic agents  ● No MetS  ● eGFR ≥ 60 ml/min/1.73m^2^  ● Predicted 10-year CVD risk < 20%  ● No clinical CVD |
| **Stage 2: Metabolic risk factors and CKD** | Individuals with metabolic risk factors (hypertriglyceridemia, hypertension, diabetes, MetS), or CKD (moderate- or high-risk)  Any of the five criteria is met:  ● Hypertriglyceridemia  ● Hypertension  ● Diabetes  ● MetS  ● Moderate-to-high-risk CKD  All criteria are met:  ● No very high-risk CKD  ● Predicted 10-year CVD risk < 20%  ● No clinical CVD |
| **Stage 3: Subclinical CVD in CKM** | Subclinical CVD among individuals with metabolic risk factors, or CKD  Any of the two criteria is met:  ● Very high-risk CKD  ● Predicted 10-year CVD risk ≥ 20%  Any of the eight criteria is met:  ●Overweight/obesity  ● Abdominal obesity  ● Prediabetes  ● Hypertriglyceridemia  ● Hypertension  ● Diabetes  ● MetS  ● Moderate-to-high-risk CKD  The criterion is met:  ●No clinical CVD |
| **Stage 4: Clinical CVD in CKM** | Clinical CVD among individuals with metabolic risk factors, or CKD  The criterion is met:  ● Clinical CVD  Any of the nine criteria is met:  ● Overweight/obesity  ● Abdominal obesity  ● Prediabetes  ● Hypertriglyceridemia  ● Hypertension  ● Diabetes  ● MetS  ● Moderate-to-high-risk CKD  ● Very high-risk CKD |

Moderate-to-high risk chronic kidney disease (CKD) was defined as an estimated glomerular filtration rate (eGFR) of 30 to <60 mL/min/1.73 m². Very high-risk CKD was defined as an eGFR of <30 mL/min/1.73 m². BMI, body mass index; CHARLS, China Health and Retirement Longitudinal Study; CKD, chronic kidney disease; CKM, cardiovascular-kidney-metabolic; CVD, cardiovascular disease; DBP, diastolic blood pressure; eGFR, estimated glomerular filtration rate; MetS, metabolic syndrome; SBP, systolic blood pressure; TG, triglyceride.

**Supplemental Table 2 SMDs before/after the IPTW**

| Variable | Unweighted_SMD | Weighted_SMD |
| --- | --- | --- |
| Age | 0.13 | 0.007 |
| Sex | -0.155 | -0.008 |
| Marital status | 0.07 | 0.006 |
| Sleeping time | -0.454 | 0.01 |
| Nap | -0.072 | 0.004 |
| Current cigarette use | -0.075 | -0.003 |
| Alcohol use_1 | -0.091 | -0.007 |
| Alcohol use_2 | -0.005 | -0.002 |
| Alcohol use_3 | 0.096 | 0.009 |
| Hb | -0.121 | 0 |
| Platelet | 0.068 | 0.004 |
| WBC | -0.014 | -0.009 |
| CRP | -0.004 | 0.002 |
| Falling down | 0.117 | 0.004 |
| Hyperuricemia | -0.014 | 0 |
| Arthritis | 0.193 | 0.002 |
| Pulmonary diseases | 0.048 | 0.003 |
| Sarcopenia | 0.064 | 0.001 |
| Digestive diseases | 0.129 | 0.002 |
| Hepatic diseases | 0.019 | 0.002 |
| Education_1 | 0.151 | 0.011 |
| Education _2 | -0.145 | -0.011 |
| Education _3 | -0.007 | 0 |
| Glucose-lowing therapy | 0.012 | 0.001 |
| Antidepressants | 0.009 | 0.001 |
| Lipid-lowering therapy | 0.01 | 0.001 |
| Antihypertensive therapy | 0.02 | 0.007 |

Hb: hemoglobin; WBC: white blood cells; CRP: C-reactive protein

**Supplemental Table 3 Missing value summary for study variables**

| Variable | Missing_N | Missing_Percent(%) |
| --- | --- | --- |
| Age | 0 | 0 |
| Sex | 0 | 0 |
| Marital status | 0 | 0 |
| Living area* | 1856 | 38.8 |
| Sleeping time | 17 | 0.36 |
| Nap | 0 | 0 |
| Current cigarette use | 0 | 0 |
| Alcohol use | 0 | 0 |
| Hb | 85 | 1.78 |
| Platelet | 85 | 1.78 |
| WBC | 85 | 1.78 |
| CRP | 0 | 0 |
| Falling down | 3 | 0.06 |
| Hyperuricemia | 0 | 0 |
| Arthritis | 0 | 0 |
| Pulmonary diseases | 4 | 0.08 |
| Sarcopenia | 0 | 0 |
| Digestive diseases | 0 | 0 |
| Hepatic diseases | 0 | 0 |
| education | 0 | 0 |
| Antihypertensive therapy | 0 | 0 |
| Lipid-lowering therapy | 0 | 0 |
| Glucose-lowing therapy | 0 | 0 |
| Antidepressants | 0 | 0 |
| Physical activity* | 2751 | 57.52 |

Hb: hemoglobin; WBC: white blood cells; CRP: C-reactive protein. *: only contained in the sensitive analysis 6.
