# Supplementary material for: Depression and risk of incident heart diseases among older adults at CKM stages 0–3: evidence from the China Health and Retirement Longitudinal Study
Source: Ann Med. 2026 Jun 26;58(1):2690815. doi: 10.1080/07853890.2026.2690815 (PMC13312823; doi:10.1080/07853890.2026.2690815)
Supplement: figure legend.docx [file IANN_A_2690815_SM2143.docx]

**Figure legend**

**Figure 1 The flow chart of screening for enrolled individuals.**

**Figure 2 The forest plot for illustrating the association between the depression and CKM stage 4/IHD** Model 1: crude model; Model 2: adjusted for age, sex, marital status, education, sleep duration, smoking, and alcohol use; Model 3: further adjusted for hemoglobin, white blood cell count, platelet count, and C-reactive protein; Model 4: further adjusted for demographic factors, laboratory parameters, clinical comorbidities and relevant medications; For models 1–4 using IHD as the outcome, the sample size was 4,783; for models using CKM stage 4 as the outcome, the sample size was 2,477.

**Figure 3 Directed Acyclic Graph (DAG) with depression as the exposure and incident heart diseases as the outcome.**

**Figure 4 The forest plot of the subgroup analysis for estimating the association between depression and IHD**

**Figure 5 RCS displaying the nonlinear relationship between the CESD score and IHD** A: based on data-driven strategy (model 4); B: based on the DAG-driven strategy (model 5. Three knots were placed at the 10th, 50th, and 90th percentiles of CESD score (values: 1, 7, 17). The reference value (OR=1) was set at the median CESD score. Shaded area represents 95% confidence interval. Most participants (90%) had CESD scores between 0 and 21.

**Figure 6 The results of the mediation effect analysis** A, B, C displayed the mediative effect of the changes in BMI, WBC, and CRP, respectively.
